# Indication of CPAP in Patients with Suspected Obstructive Sleep Apnea, Based on Clinical Parameters and a Novel Two-Channel Recording Device (ApneaLink): A Pilot Study

**DOI:** 10.1155/2012/346181

**Published:** 2012-10-02

**Authors:** Carlos Alberto Nigro, Eduardo Dibur, Sofía Grandval, Facundo Nogueira

**Affiliations:** ^1^Sleep Laboratory, Pneumonology Unit, Hospital Alemán, Avenue Pueyrredón 1640, Buenos Aires C1118AAT, Argentina; ^2^Sleep Laboratory, Instituto Argentino de Investigación Neurológica, Uruguay 840, Buenos Aires C1015ABR, Argentina

## Abstract

*Objective.* To evaluate the accuracy and reliability of the medical decision based on the results of the hand scoring from a two-channel recording device (ApneaLink) plus clinical data for the prescription of a CPAP assay in patients with suspected OSA. *Methods.* 39 subjects were assessed in the sleep laboratory with polysomnography and ApneaLink. The patients completed the Epworth sleepiness scale and a clinical history. Two blinded independent observers decided to prescribe CPAP according to the results of the PSG (gold standard, observer A), ApneaLink (alternative method, observer B), and the clinical parameters. Sensitivity and specificity of observer B on the indication of CPAP were calculated. The interobserver agreement for the indication of CPAP was assessed using kappa statistics. *Results.* 38 subjects were included (26 men, mean age 47.5, mean RDI 28.7, mean BMI 31.4 kg/m^2^). The prevalence of OSA was 84%. The sensitivity and specificity of observer B to initiate a CPAP trial were 90.6% and 100%, respectively. The interrater agreement for the prescription of CPAP was good (kappa: 0.75). *Conclusion.* This study has shown that the use of ApneaLink plus clinical data has made it possible to indicate CPAP reliably in most patients with high-clinical pretest for OSA.

## 1. Introduction

Obstructive sleep apnea (OSA) is a major health problem due to its prevalence rates of 2–4% in middle-aged people [1] and significant morbidity [2, 3] and mortality [4] reported in patients with this condition. Continuous positive airway pressure (CPAP) is an effective treatment commonly prescribed for symptomatic patients with obstructive sleep apnea. It is costeffective [5] and reduces daytime sleepiness [6], rates of motor vehicle accidents [7], and blood pressure [8]. The American Thoracic Society [9] and the American Academy of Sleep Medicine [10] recommend supervised polysomnography (PSG) in the sleep laboratory over two nights for the diagnosis of obstructive sleep apnea and initiation of CPAP. This approach to a highly prevalent condition results in inevitable discrepancies between the demand for services and the current capacity of sleep laboratories [11]. Because the treatment of sleep apnea provides many benefits to patients and society, it is imperative to develop strategies that are less complex than the traditional approach based on PSG to expedite the diagnosis and treatment of patients with OSA.

The ApneaLink is a two-channel screening tool for sleep apnea. The device consists of a nasal cannula attached to a small case that houses a pressure transducer and a pulse oximeter. It is held in place by a belt worn around the user's chest. It has good potential as a simple screening device, particularly because it allows the manual review and scoring of the raw data. There are several reports which have evaluated the performance of the automatic analysis of nasal pressure from the ApneaLink device against polysomnography to diagnosis sleep apnea [12–15]. Furthermore, we have recently demonstrated that the manual analysis of data using an AHI (ApneaLink) and an RDI (PSG) ≥5 as criteria for OSA has showed a sensitivity and specificity nearly 90% [16]. Thus, the objective of this study was to evaluate the accuracy and reliability of the medical decision based on the results of the hand scoring from a two-channel recording device (ApneaLink) plus clinical data (excessive daytime sleepiness, cardiovascular comorbidities, and type 2 diabetes) for the prescription of CPAP in patients with suspected OSA.

## 2. Methods

### 2.1. Patients

Forty consecutive patients referred to the Hospital Alemán Sleep Laboratory for the investigation of possible OSA were invited to participate in the study. The inclusion criteria were patients of both sexes with suspected OSA (snoring with/without other symptoms such as apneas referred by someone and/or somnolence), age equal to or over 18 years old, and informed consent. The patients who used oxygen, CPAP or some modality of noninvasive mechanical respiratory assistance during PSG and those subjects suspected of having congestive heart failure, neuromuscular disease, insomnia, parasomnias, periodic limb movement disorder, circadian rhythm disorders, or narcolepsy by medical history were excluded from the study. The polysomnographies with artefacts in EEG or respiratory channels (airflow, thoracoabdominal movements and SO_2_) that did not allow the reading of the sleep stages or the respiratory events or with less than 180 minutes of total sleep time and the recordings of ApneaLink with less than 4 h of manual evaluation period were not considered for analysis.

### 2.2. Study Protocol

The subjects were assessed in the sleep laboratory simultaneously with polysomnography (PSG) and ApneaLink. Prior to the polysomnography, the patients completed the Spanish version of the Epworth sleepiness scale (ESS) [17] and a clinical history (see Table 1). Two independent blinded observers reviewed the results from the polysomnography (observer A, ED), the ApneaLink (observer B, CN), and the clinical data. Then, we simulated a situation in which both observers could initiate treatment with CPAP in patients with a diagnosis of OSA based on the results from the polysomnography (observer A), ApneaLink (observer B), and clinical parameters. The reference method to prescribe CPAP arose from symptoms and the respiratory disturbance index (RDI) from PSG (observer A). On the other hand, the alternative method to prescribe CPAP was based upon the symptoms and the apnea/hypopnea index of ApneaLink (observer B). Observer A decided to prescribe CPAP in those patients who showed an RDI  ≥15 regardless of the patients' symptoms or associated comorbidities or when the RDI was between 5 and 15 in a patient with excessive daytime sleepiness (Epworth >10) or any of the following comorbidities: hypertension, cardiac arrhythmias, coronary heart disease, cerebral vascular disease, or type 2 diabetes [18, 19]. On the other hand, observer B decided to initiate a CPAP trial in those patients with any of the following conditions: (1) ApneaLink compatible with moderate to severe OSA (AHI ≥15) or (2) ApneaLink consistent with mild to moderate OSA (AHI ≥5–<15) plus the presence of excessive daytime sleepiness (Epworth  >10) or any comorbidities.

### 2.3. Measurements

#### 2.3.1. Polysomnography

All the patients underwent overnight polysomnography with a computerised polysomnographic system (Mini PC, Akonic, Buenos Aires, Argentina or Harmonie, Stellate Systems, Canada) (F4/A1, C4/A1, and O2/A1), bilateral electrooculogram, submental electromyogram, bilateral leg electromyogram, and electrocardiogram. Airflow was measured by nasal pressure and oral thermistor; respiratory effort was assessed by a thoracoabdominal piezoelectric belt, and oxygen saturation (SO_2_) was recorded using a finger probe (Nonin, Plymouth, Minn, USA). The polysomnographies were registered from 10.30–11.30 PM to 05-06 AM. On the day of the study, the patients were given the following instructions: (1) to avoid napping and not to drink alcohol or beverages with caffeine (coffee, tea, and cola drinks); (2) to continue the usual medication; (3) to eat supper between 8.30 and 9.30 PM; (4) to report to the sleep lab between 10.30 and 11.30 PM.

**PSG Analysis**

PSG reading was performed manually by two widely experienced medical staff members who were blind to the operator that analysed the ApneaLink. The sleep stages were analysed in 30 s epochs according to international criteria [20]. The arousals were identified following the American Sleep Disorder Association recommendations [21]. The analysis of apneas, hypopneas, and respiratory effort related arousals (RERAs) were in agreement with the international criteria [22, 23]. The following definitions were used.

- Respiratory disturbance index (RDI): number of apneas plus hypopneas plus RERAs per hour of sleep [24].
- OSA was defined as an RDI ≥5.
- Severity of OSA: mild = RDI ≥5–<15; moderate = RDI ≥15–<30; severe RDI ≥30 [25].

#### 2.3.2. ApneaLink

ApneaLink records patient respiratory nasal pressure and blood oxygen saturation during sleep. The nasal pressure is measured directly at the nostrils and is not linear to the patient's breathing flow. In order to reestablish this linearity, a mathematical formula is used for linearizing the nasal pressure. The linearization ensures that even the smallest changes in the patient's breathing flow are recorded and evaluated validly [26]. The blood oxygen saturation and pulse rate are measured using the finger pulse sensor and the pulse oximeter (XPod, Nonin). The XPod module has motion-tolerant software that minimizes the likelihood of motion artifact being misinterpreted as good pulse quality. The ApneaLink device operates on battery power, has a sampling rate of 100 Hz (airflow), 1 Hz (oxygen saturation) and a 20-bit signal processor. The internal memory storage is 15 MB, which allows for approximately 10 hours of data collection. During the night of laboratory evaluation, subjects also wear an ApneaLink device. The nasal cannula used by the patients during the study is attached to a “*T*” connector leading to a pressure transducer, allowing for the simultaneous recording of the flow signal by the ApneaLink device and the PSG system.

**ApneaLink Analysis**

Using the ApneaLink software (version 9.00) installed in a PC, one blind observer (CAN) who was independent from the results of the PSG performed the automatic analysis first, and then, he did the full manual correction.

(i)  Automatic Scoring: the ApneaLink default setting for apnea was used. An apnea was defined as a decrease in airflow by 80% from baseline for at least 10 seconds. The ApneaLink default maximum apnea duration was set at 80 seconds. A hypopnea was defined as a decrease in airflow ≥30% from baseline for at least 10 seconds plus oxygen desaturation ≥3%. The ApneaLink default maximum hypopnea duration was set at 100 seconds. The apnea/hypopnea index was calculated as the number of apneas/hypopneas per hour of automatic evaluation period (AHI-a).
(ii)  Manual Scoring: once the ApneaLink program had carried out the automatic analysis, the results were revised in 3 or 5 min epochs and, when appropriate, manually corrected by a physician trained (CAN) in the reading of polysomnography. If required, the operator could edit or delete events or insert new ones. Likewise, it was possible to include or exclude sectors of the recording for their analysis. Apnea was defined as the absence of airflow lasting ≥10 s from baseline for at least 10 seconds. Hypopnea was considered when a reduction of airflow ≥10 s from baseline plus oxygen desaturation ≥3% or evidence of autonomic arousal was identified at the end of hypopneas or when only a reduction of airflow ≥50% was observed. We used an increase in the pulse rate of at least five beats per minute as criterion for autonomic arousal [27, 28]. The manual apnea/hypopnea (AHI-m) was calculated as the number of apneas/hypopneas per hour of evaluation period. The positive ApneaLink criteria used in this study were an AHI-m ≥5.

### 2.4. Statistical Analysis

To assess if the study variables had a normal distribution a frequency histogram and the Kolmogorov-Smirnov test were performed. Thus, when the distribution was normal, the mean and standard deviation was reported. Instead, the median and the percentiles 25–75% were used if the distribution was not normal. The degree of association among the manual ApneaLink apnea/hypopnea index variables (AHI-m) and the respiratory disturbance (RDI) from polysomnography was evaluated by the Pearson correlation coefficient (*r*). The nature and extent of the disagreement between the RDI and the AHI-m were assessed by means of a Bland and Altman plot. Accuracy of observer B on the indication of CPAP was assessed by the receiver operating characteristics (ROC) curve. Sensitivity, specificity as well as positive and negative likelihood ratio (LR+, LR−) were calculated. The interobserver agreement for the indication of CPAP and manual scoring of ApneaLink was assessed by kappa statistics. The statistic analysis was made with a commercially available software programme (MedCalc Software, Version 11.3, Mariakerke, Belgium. 

## 3. Results

Out of the 40 patients who were invited into the study, 39 gave informed consent and one patient was ruled out due to an ApneaLink signal with frequent artifacts by finger clip probe disconnections. Thus, 38 subjects provided acceptable data for the final analysis. The patient characteristics are shown in Table 2. Men represented 68.5% of the study sample. The prevalence of OSA in the sample study was 84.2%. 23.7% had mild OSA and 60.5% of subjects had moderate to severe sleep apnea (*P* < 0.01).

**Figure 1:**
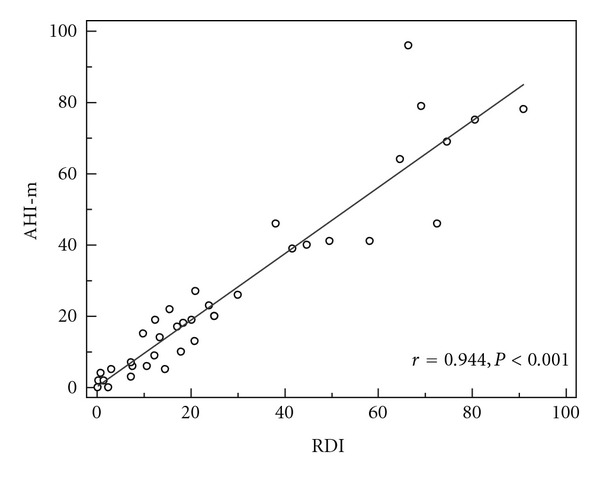
Scatter diagram and regression line between the manual AHI from Apnealink (AHI-m) and the respiratory disturbance index of the polysomnography (RDI).

**Figure 2:**
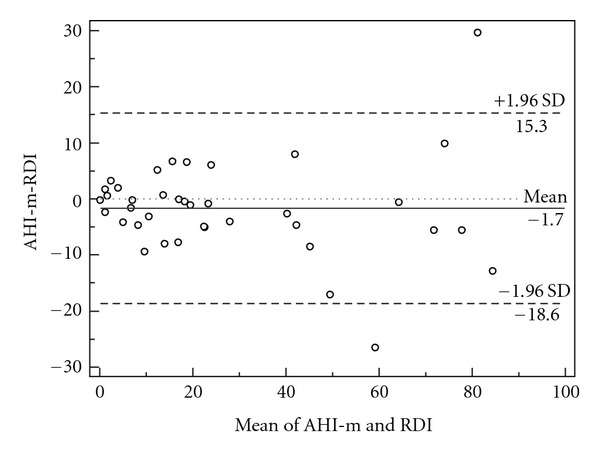
Bland-Altman plot of manual ApneaLink apnea/hypopnea index (AHI-m) and the respiratory disturbance index (RDI) from PSG.

There was a strong correlation between the AHI-m of ApneaLink and the RDI from polysomnography (*r* = 0.944, *P* < 0.001) (Figure 1). The mean difference between the manual scoring from ApneaLink (AHI-m) and the PSG (RDI) was −1.7 ± 8.6 (Figure 2). 

The sensitivity and specificity of observer B to prescribe CPAP were 90.6%, and 100% respectively (see Table 3). The false negative cases (FN) were three patients with mild to moderate OSA (RDI: 7.3–21) who showed a total sleep time and sleep efficiency lower than the true positive (TP) patients. In addition, the FN had a higher proportion of RERAs than the TP cases (see Table 4). The interrater agreement for the prescription of CPAP was good (*k* = 0.75). Similarly, the agreement between observers for the manual analysis of ApneaLink in a subgroup of 15 patients selected at random was very good (*k* = 0.81). 

## 4. Discussion

These data suggest that an experienced physician using clinical parameters and the results of a device that measures airflow by nasal pressure and oximetry (ApneaLink) might prescribe a CPAP trial with reasonable accuracy in subjects with suspected OSA who had indication of initiating treatment with CPAP by medical history and polysomnography. This theoretical model to prescribe a CPAP trial had a sensitivity of 90% and a specificity of 100%. Furthermore, we observed a good agreement between observers for prescribing a CPAP proof (*k* = 0.75). The main drawback of this approach could be that 10% of patients with suspected OSA, who could have benefited with CPAP therapy, were excluded from the treatment because they did not meet the ApneaLink or clinical criteria to initiate a CPAP trial. In these false negative cases, it would have been necessary to indicate a polysomnography with the consequent increase of the costs and delay in the diagnosis and treatment. On the other hand, no patient initiated a trial of CPAP unnecessarily since the specificity of this approach was of 100%. These results are consistent with previous studies. Mulgrew et al. [29] showed that in the initial management of patients with a high probability of obstructive sleep apnea, polysomnography did not confer any advantage over the ambulatory approach based on oximetry and auto-CPAP in terms of diagnosis, CPAP titration, and clinical outcomes. A recent simulation study showed that two experts using a comprehensive sleep history without a sleep study could have reliably initiated CPAP in 52% of the patients with suspected OSA; this results in significant cost reduction [30]. Four prospective randomized studies that used respiratory polygraphy [31, 32] or level 4 sleep devices [33, 34] have shown that the clinical course of patients with OSA diagnosed and treated at home with CPAP was similar to the group of patients who underwent polysomnography with CPAP titration in the sleep laboratory. Finally, we observed that the combination of clinical data and oximetry had a high accuracy for prescribing CPAP in patients with suspected OSA compared with polysomnography [35]. Based on our results, in patients with high clinical pretest for OSA, we currently diagnose and prescribe CPAP according to the results of the ApneaLink conducted at home-plus-clinical parameters.

Our study has some limitations. A study to support a treatment decision requires a greater number of patients. This is a theoretical analysis of the usefulness of ApneaLink and the clinic history to diagnose and indicate a CPAP trial in patients with OSA. In order to evaluate the accuracy of this strategy in real life, it is necessary to conduct a randomized study to draw conclusions that are more valid. As a new approach for clinical decision making, it is necessary to look at its influence on long-term outcomes like compliance of CPAP and possible effect on cardiovascular comorbidities. In addition, the use of these diagnostic tools for the indication of CPAP requires expertise in the diagnosis of sleep disorders and training in the analysis of the ApneaLink. 

In conclusion, this study has shown the use of an approach based on ApneaLink, and clinical data has made it possible to indicate CPAP reliably in most patients with high clinical pretest for OSA. This approach could be used in situations where access to sleep studies is not possible, with the consequent reduction of costs and rapid initiation of treatment with CPAP, especially in the most severe forms of OSA. 

## Figures and Tables

**Table 1:** Clinical parameters.

(1) Occupation
(2) Alcohol consumption
(3) Smoking
(4) Body mass index (BMI)
(5) Berlin questionnaire
(6) Epworth sleepiness scale
(5) Comorbidities
Chronic obstructive pulmonary disease
Asthma
Pulmonary fibrosis
Pulmonary hypertension
Coronary arterial disease
Hypertension
Cardiac arrhythmia
Cardiac failure
Cerebrovascular disease
Diabetes mellitus
Hypothyroidism
Acromegaly
Menopause
Nasal obstruction, rhinitis
Neuromuscular diseases
Other diseases
(6) Medications
(7) Other sleep disorders
Sleep deprivation
Restless legs syndrome
Narcolepsy
Insomnia
Other sleep disorders

**Table 2:** Patient characteristics.

Patient number	38
Age (years)	47.5 ± 15.2
Men	26 (68.5)
BMI (body mass index—kg/m^2^)	31.4 ± 7.4
Prevalence of OSA (%)	
RDI ≥ 5	32 (84.2)
Severity of OSA (%)	
RDI ≥5–<15	9 (23.7)
RDI ≥15–<30	10 (26.3)
RDI ≥ 30	13 (34.2)
PSG	
TRT (total recording time: min.)	362.9 ± 22.7
TST (total sleep time: min.)	317.2 ± 38.4
TWT (total wakefulness time: min.)	47.6 ± 36.5
SE (sleep efficiency)	0.87 ± 0.1
TNREM (min.)	283.2 ± 43
TREM (min.)	34 ± 29.1
RDI (respiratory disturbance index)	28.7 ± 7.3
Comorbidities	
Hypertension	10 (26)
Coronary heart disease	3 (8)
Cerebrovascular ischemia	1 (3)
Arrhythmia	6 (16)
Asthma	1 (3)
Allergic rhinitis	11 (29)

Data are presented as mean ± SD, or *n* (%). OSA: obstructive sleep apnea. TNREM: total stages 1 + 2 + 3 + 4; TREM: total amount of REM sleep.

**Table 3:** Sensitivity, specificity, positive and negative likelihood ratio, and predictive value of ApneaLink plus clinical data for the prescription of CPAP.

AUC-ROC (SE)	Sensitivity (CI 95%)	Specificity (CI 95%)	+LR	−LR	+PV (CI 95%)	−PV (CI 95%)
0.953 (0.026)	90.6 (75–98)	100 (54.1−100)		0.094	100 (87.7−100)	67 (30−92.5)

AUC-ROC: area under the ROC curve; SE: standard error; + and −LR: positive and negative likelihood ratio; + and −PV: positive and negative predictive value.

CI 95%: confidence interval 95%.

**Table 4:** Characteristics of false negative patients.

	False negative	True positive	*P*
Number	3	29	
Total sleep time	253.4 ± 11.8	329.2 ± 29	0.0003
Sleep efficiency	0.70 ± 0.06	0.90 ± 0.08	0.013
RDI	15.3 ± 7.1	35.7 ± 25.7	0.009
RERAs index	4.5 ± 4.2	0.98 ± 2	0.012

*Values are expressed as mean ± SD. RERAs index: respiratory-effort-related arousals index.
